# The toxoplasma-host cell junction is anchored to the cell cortex to sustain parasite invasive force

**DOI:** 10.1186/s12915-014-0108-y

**Published:** 2014-12-31

**Authors:** Marion Bichet, Candie Joly, Ahmed Hadj Henni, Thomas Guilbert, Marie Xémard, Vincent Tafani, Vanessa Lagal, Guillaume Charras, Isabelle Tardieux

**Affiliations:** Department of Cell Biology of Host-Pathogen Interactions, Inserm U1016, Institut Cochin, 22 Rue Méchain, 75014 Paris, France; Department of Cell Biology of Host-Pathogen Interactions, Cnrs UMR8104, 22 Rue Méchain, 75014 Paris, France; Department of Cell Biology of Host-Pathogen Interactions, Université Paris Descartes, Sorbonne Paris Cité, 22 Rue Méchain, 75014 Paris, France; Department of Cell and Developmental Biology, London Centre for Nanotechnology, University College London, 17-19 Gordon Street, WC1H 0AH London, UK

**Keywords:** Cortical actin, Host cell Invasion, Kinematics, Toxofilin, Toxoplasma

## Abstract

**Background:**

The public health threats imposed by toxoplasmosis worldwide and by malaria in sub-Saharan countries are directly associated with the capacity of their related causative agents Toxoplasma and Plasmodium, respectively, to colonize and expand inside host cells. Therefore, deciphering how these two Apicomplexan protozoan parasites access their host cells has been highlighted as a priority research with the perspective of designing anti-invasive molecules to prevent diseases. Central to the mechanism of invasion for both genera is mechanical force, which is thought to be applied by the parasite at the interface between the two cells following assembly of a unique cell-cell junction but this model lacks direct evidence and has been challenged by recent genetic studies. In this work, using parasites expressing the fluorescent core component of this junction, we analyze characteristic features of the kinematics of penetration of more than 1,000 invasion events.

**Results:**

The majority of invasion events occur with a typical forward rotational progression of the parasite through a static junction into an invaginating host cell plasma membrane. However, if parasites encounter resistance and if the junction is not strongly anchored to the host cell cortex, as when parasites do not secrete the toxofilin protein and, therefore, are unable to locally remodel the cortical actin cytoskeleton, the junction travels retrogradely with the host cell membrane along the parasite surface allowing the formation of a functional vacuole. Kinetic measurements of the invasive trajectories strongly support a similar parasite driven force in both static and capped junctions, both of which lead to successful invasion. However, about 20% of toxofilin mutants fail to enter and eventually disengage from the host cell membrane while the secreted RhOptry Neck (RON2) molecules are posteriorally capped before being cleaved and released in the medium. By contrast in cells characterized by low cortex tension and high cortical actin dynamics junction capping and entry failure are drastically reduced.

**Conclusions:**

This kinematic analysis newly highlights that to invade cells parasites need to engage their motor with the junction molecular complex where force is efficiently applied only upon proper anchorage to the host cell membrane and cortex.

**Electronic supplementary material:**

The online version of this article (doi:10.1186/s12915-014-0108-y) contains supplementary material, which is available to authorized users.

## Background

Toxoplasmosis is a worldwide spread zoonosis caused by the protozoan Apicomplexa parasite *Toxoplasma gondii* that imposes serious economic loss in livestock. It is also a concern in human health since about a third of the population is thought to silently carry parasites, which under immunosuppressive conditions, revert to replicative parasites called tachyzoites. Subsequent uncontrolled expansion of the tachyzoite population is commonly responsible for cerebral, cardiac and pulmonary life-threatening diseases. Because tachyzoites only multiply in a parasitophorous vacuole (PV) that derives from the host cell plasma membrane (PM) invagination at the time of entry [1], tachyzoite invasiveness is thus a primary determinant of *Toxoplasma* infection outcome. Such strict dependence on host cells has impelled decades of research to decipher the molecular mechanisms of the invasion event and eventually to design anti-invasion strategies as pharmacological or immunological approaches to control infection and to prevent diseases [2]. Other Apicomplexa zoites, in particular the etiological agents of malaria, that is, *Plasmodium spp* parasites*,* invade host cells and use a similar strategy to this end; therefore, the long-lasting interest in host cell invasion and the pressing need to progress in this research go much beyond *Toxoplasma*.

While the active participation of a membrane-associated contractile system of *Apicomplexa* zoites during host cell entry was emphasized in the 1980s [3–5] and later assigned to a conserved actin-myosin (MyoA)-based force [6–8], a contribution of the host cell through cortical actin dynamics has been more recently unmasked [9,10]. To establish an intimate contact with a permissive host cell, zoites secrete at their apical pole a protein complex from vesicles called the rhoptries (RhOptry Neck (RON) complex), that assembles as a ring into the host cell PM and beneath [11–14] and that connects with *de novo*-nucleated host cell actin filaments [9]. Therefore, the current model specifies that zoites trigger the transient buildup of a unique tight interface, called a junction, between the two cells that serves as a door of entry and that seems optimally anchored to the host cell cortical actin cytoskeleton to act as a traction site for the parasite motor-based force production [2,15]. The *T. gondii* rhoptry protein toxofilin that loosens the host cell cortical actin meshwork at the onset of invasion has been proposed to promote local availability of actin monomers for actin assembly at the junction [16].

Although the recent localization of actin juxtaposed to the RON-positive ring in *Plasmodium* merozoite invading an erythrocyte [17–19] is in line with the zoite motor force scheme, such observation has not been confirmed for *T. gondii* tachyzoites. In addition, the ‘force transmitting’ function of two molecules that back up the model by acting as physical bridges between the RON ring and the parasite motor, namely AMA1 and the glycolytic enzyme aldolase [20,21], has been recently questioned [22–24] while no other potential linkers to fulfill the role have been identified. Moreover, an actin/myoA-independent mode of entry has been evoked as an alternative strategy since *T. gondii* tachyzoites devoid of actin or myoA showed unexpected residual gliding and invasive capabilities [25,26]. Finally, recent theoretical modeling of erythrocyte invasion by *Plasmodium* merozoite highlighted the possibility that host cell membrane projections induced by the parasite could promote its firm positioning on the red blood cell surface as well as its subsequent internalization, thereby shifting the model towards collaboration for force production between the two partners [27]. While traction forces between *Plasmodium* sporozoites and substrates have been measured using reflection interference contrast and traction force microscopy [28], no direct evidence for a traction force exerted by the zoite at the junction has ever been demonstrated for *Plasmodium* or *Toxoplasma*. These major flaws of the model are in large part due to the difficulty of tracing actin/myoA dynamics or junction components in live cells during the high-speed entry process, which lasts tens of seconds and involves tiny amounts of molecules.

In this context, we decided to inspect in detail the force origin and features powering parasite entry into the host cell by simultaneously tracking the tachyzoite apex, the tachyzoite-cell junction and the host PM during the penetration event. To this end, we used tachyzoites expressing a fluorescent functional RON2 (RHΔ*Ku80:Ron2mCherry*) [22] that marks the junction site being the RON complex core component, which spans the host cell plasma bilayer [12]. Kinematic analysis of parasite pre-invasive and invasive behaviors revealed a major scenario that includes (1) a minimal impulse of a few microns per second speed followed by (2) a brief decrease in motion coinciding with RONs release and insertion into the host cell PM, and then (3) a rotational progression at a few tens of microns per second into the nascent PV while the junction remains *quasi* stationary. However, when parasites encounter some higher resistance that impedes progression or when the junction is not properly anchored, the latter flows retrogradely along the parasite surface and the host PM eventually encloses the zoite in a growth permissive PV. Kinematic measurements during host cell entry strongly argue for a similar parasite driven force in static and capped junctions. Importantly, when the junction is not well anchored to the host actin cortex the secreted RON2 molecules are displaced without the PM to the posterior pole and are then shed from the zoite, which as a consequence disengages from the host cell PM. Accordingly, constitutively blebbing cells that display low cortex tension and high cortical actin dynamics provide the optimal conditions for stable junction and successful invasion. Collectively, these data demonstrate that the zoite applies a motor force onto the RON2-containing junction that leads to invasion when the latter is properly anchored to the host cell cortex.

## Results

### A minimum impulse of the tachyzoite is typically associated with penetration into host cells

**Figure 1:**
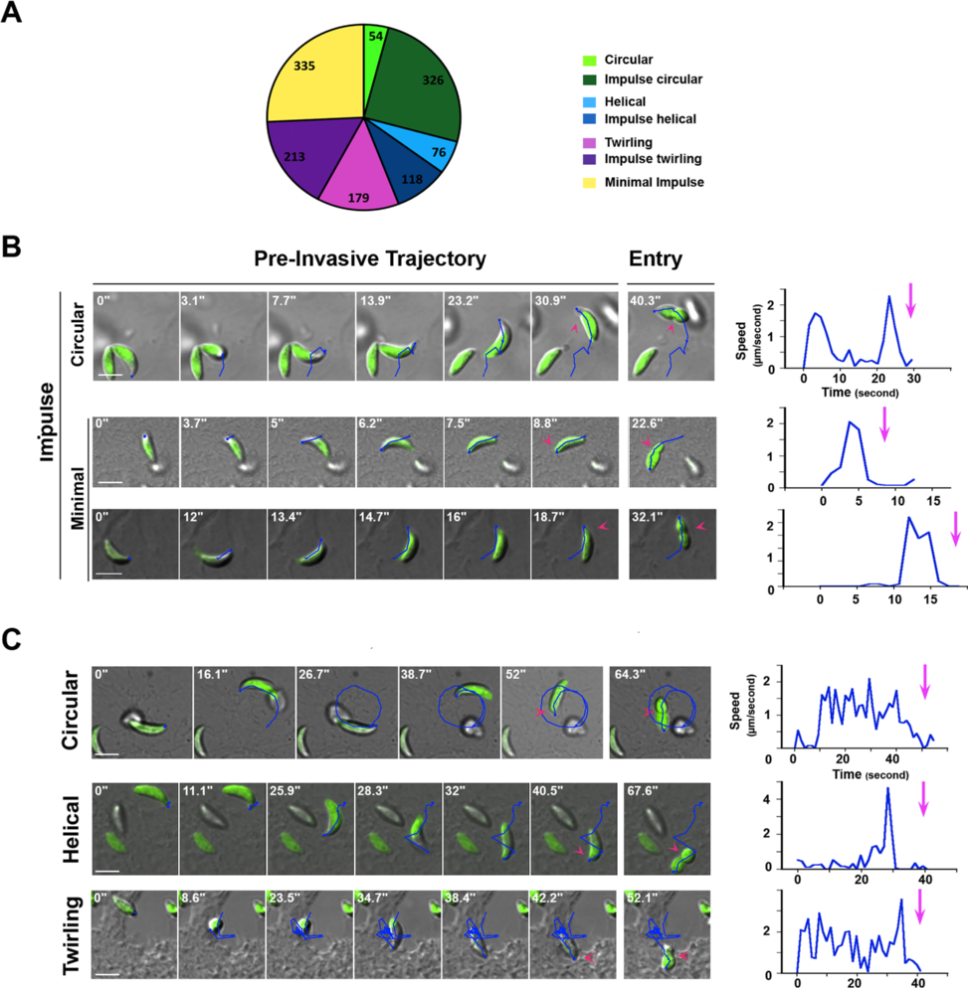
**Pre-invasive trajectories of GFP-expressing tachyzoites. (A)** Pie graph showing the distribution of each type of pre-invasive motion, the absolute numbers are indicated, **(B-C)** DIC-GFP merged time lapses showing tachyzoite **(B)** impulse circular motion on top of HFF cells (top panel), and minimal impulse on top of Ptk-1 (medium panel) and NRK (bottom panel) cells, and **(C)** circular gliding onto NRK cells (top panel), helical gliding onto Ptk-1 cells (second panel) and twirling gliding onto M2 cells (bottom panel). The blue line reconstitutes the trajectory of the parasite apex; pink arrows define the junction; the last frame of each time lapse attests to tachyzoite entry; graphs show the tachyzoite speed over time and the pink arrows mark the starting point of the penetration event; all scale bars: 5 μm. DIC, differentia interference constrast; HFF, human foreskin fibroblasts; NRK, normal rat kidney fibroblasts; Ptk-1, rat kangaroo kidney epithelial cells.

Pioneer video-microscopic and kinematic studies [29] revealed three main types of *T. gondii* tachyzoite substrate-dependent movements, all associated with body rotation along the long axis. The helical and circular types of gliding ensure forward progression and depend on functional actin, unlike the stationary twirling motion [4,29,30]. The helical type has been proposed to also support cell invasion [30] based on the observation that tachyzoites often glide toward host cells and invade them. Interestingly, this helical gliding is also characteristic of tachyzoite motility in a three-dimensional environment [31]. In order to check whether gliding motion increases the frequency of penetration events independently of the need to find a host cell, we performed time-lapse spinning-disk confocal imaging of parasites incubated with monolayers of epithelial or fibroblastic cells, and we analyzed the pre-invasive behavior of tachyzoites. Under these conditions, the probability of contact with a potential host cell was P = 1 for every parasite. We used fluorescent parasites that stably express GFP, which distributes in the whole cell, and we recorded spatio-temporal xyt coordinates of the parasite apex to reconstruct parasite trajectories prior to the penetration process using ImageJ software and the ‘ManualTracking’ plugin. For the vast majority of invasion events (n = 992/1,301, Figure 1A,B), we found that immobile parasites initiated a pivoting movement prior to entry that we refer to as an ‘impulse.’ This impulse could be minimal without being associated with specific motion but it was often followed by a short helical twist or by a partial circular motion (Figure 1A,B and Additional file 2: Movie 1). Apart from these impulses, circular and helical types of forward gliding, sometimes even within the same sequence, as well as twirling rotation were also found associated with cell penetration (Figure 1C, Additional file 3: Movie 2; Additional file 4: Movie 3: Additional file 5: Movie 4). The pre-invasive trajectories (blue) and the time of entry diagnosed by the first detection of body constriction (pink arrowhead) are indicated on the time lapses. The last frames show the ongoing entry process and, thus, validate the pre-invasive status of the trajectories (see the pink arrowhead when parasites are half way in). Graphs of the parasite speed prior to cell penetration reveal that in all cases, parasite speed reached a few microns per second (Figure 1A-C, the pink arrow points to the time at which entry starts). In line with a motile behavior facilitating cell invasion, we observed an increase in the rate of invasion of epithelial or fibroblastic monolayers when the motility of GFP-expressing tachyzoites was stimulated with the ‘Motility Enhancer compound, 130038 (1.4- to 1.7-fold, three independent 15 minute invasion assays, n = 50.000 fluorescence activated cell sorting (FACS) analyzed cells per assay and per condition, in duplicates), confirming previous reports [32]. Collectively, these data suggest that a minimal impulse acts to prepare tachyzoites for invasion, possibly by promoting apex positioning, and/or rhoptry fusion [33] to optimize subsequent release of the RON complex into the PM. Accordingly, this minimal movement may relate to the marked apical reorientation step that was initially recognized in the sequence of red blood cell invasion by *Plasmodium* merozoites [34].

### A major burst of RON2 molecules traffics to the conoid tip and inserts into the host cell PM in a few seconds preceding penetration

**Figure 2:**
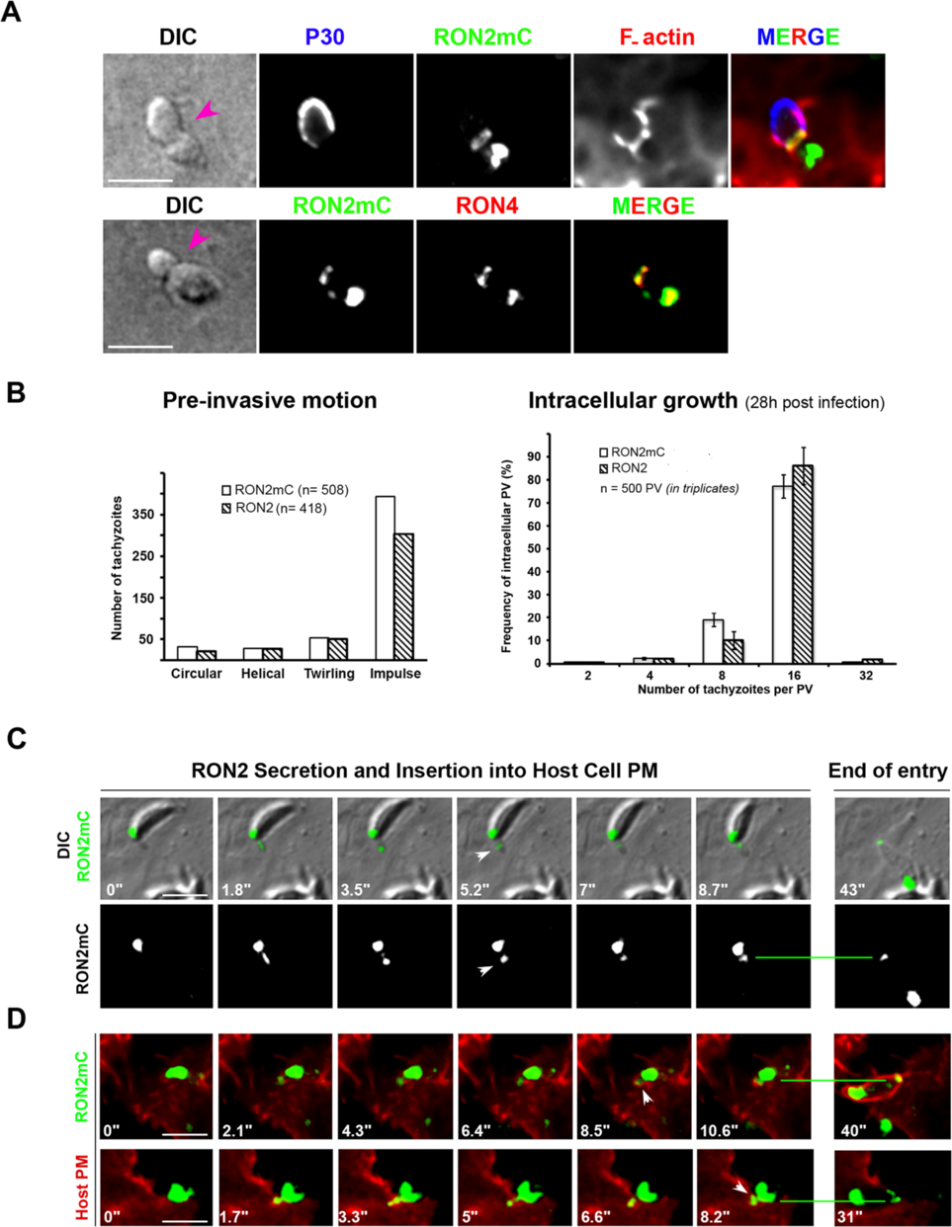
**RON2mCherry is properly targeted to rhoptries and released to the junction during invasion and RON2mC tachyzoites have normal pre-invasive and intracellular growth properties. (A)** Confocal imaging of two RON2mC tachyzoites in the course of entering into HeLa cells; (top panel). Cells were labeled for the surface-exposed P30 *T. gondii* protein (blue) prior to cell permeabilization and the host cell F-actin (red) after TX-100 cell permeabilization, (bottom panel) for the total RON4 protein a subset of which being localized at the junction (green) and serving as a marker (bottom panel); the pink arrowheads point to the junction; note the overlap between RON4 and RON2mC in the rhoptry compartments and the junction as well as the recruitment of host cell F-actin beneath the RON-formed junction. **(B)** Histograms comparing tachyzoites expressing untagged RON2 (RON2) or RON2-mCherry (RON2mC) tagged for: (left panel), the frequency of each pre-invasive behavior and (right panel) the frequency of PV containing 4, 8, 16 or 32 tachyzoites 28 hours post infection. **(C, D)** Time lapses showing RON2 trafficking to the tip of the extruded conoid, followed by RON2 assembly into **(C)** the HFF cell PM (DIC and the RON2mC (pseudo-colored in green) merged signals, **(D)** the HeLa Myr-Palm-GFP- (red, top panel) and HeLa GFP-PH-PLCδ-labeled PMs (red, bottom panel) when indicated by a white arrowhead, the green line points to the position of the junction at the early and ending (right frame) times of cell penetration; all scale bars: 5 μm. DIC, HeLa, human epithelial cervical cancer cells; HFF, human foreskin fibroblasts; Myr-Palm, myristoylated and palmitoylated; PM, plasma membrane; PV, parasitophorous vacuole; RON, RhOptry Neck.

During gliding motion, tachyzoites typically extrude their retractile apical conoid, otherwise enclosed in the cell cytoplasm [35]. In addition to promoting the release of micronemal molecules at the zoite surface, conoid repositioning might also facilitate rhoptry content release in the presence of potential host cells. The use of RON2-mCherry (RON2mC)-expressing tachyzoites that properly target RON2mC to the rhoptries and to the junction (Figure 2A), invade with similar kinetics (that is, 24.3 ± 7.8 seconds, for n = 70 RON2 tachyzoites and 26.3 ± 8.1 seconds for n = 68 RON2mC tachyzoites, *P* <0.001) and display similar pre-invasive and growth behaviors as the RON2-expressing tachyzoites (Figure 2B), allowed us to monitor in real time (1) the transit of RON2 molecules, likely with the other RON complex members within the rhoptries duct, and (2) the subsequent insertion of the RON2-ring in the host cell PM. During the movement that preceded all invasion events, we detected a major burst of RON2 secreted molecules trafficking to the tip of the conoid in the form of a dot that rapidly organizes into the host cell PM plane (Figure 2C, see white arrowheads, the RON2mC has been pseudo-colored in green). The whole process lasted a few seconds (3.2 ± 0.4 seconds, n = 45) while it appeared slightly longer when host cells expressed at the PM the PIP2-binding PH domain of PhosphoLipaseC (PLCδ) or a myristoylated and palmitoylated (Myr-Palm)-modified GFP (5.1 ± 0.8 seconds, n = 36) (Figure 2D, Additional file 5: Movie 4). No such RON2 trafficking was ever observed in gliding tachyzoites on FCS- or BSA-coated coverslips even when conoid extrusion was induced by an exposure to ethanol or calcium ionophore as described in [36]. On several occasions, while two parasites were similarly gliding on top of the same cell, both extruding their conoid, one secreted RON2 and subsequently entered in the host cell while the other did not release RON2 but remained extracellular (see Additional file 7: Movie 6). Collectively, these data support the view that (1) one or several distinct molecular interactions with the host cell surface specifically trigger the RON complex secretion, and (2) a one-shot secretion is used for junction assembly into the host cell PM.

### The RON2-positive junction is capped with the host PM to the parasite rear pole when forward motion into the cell is interrupted

**Figure 3:**
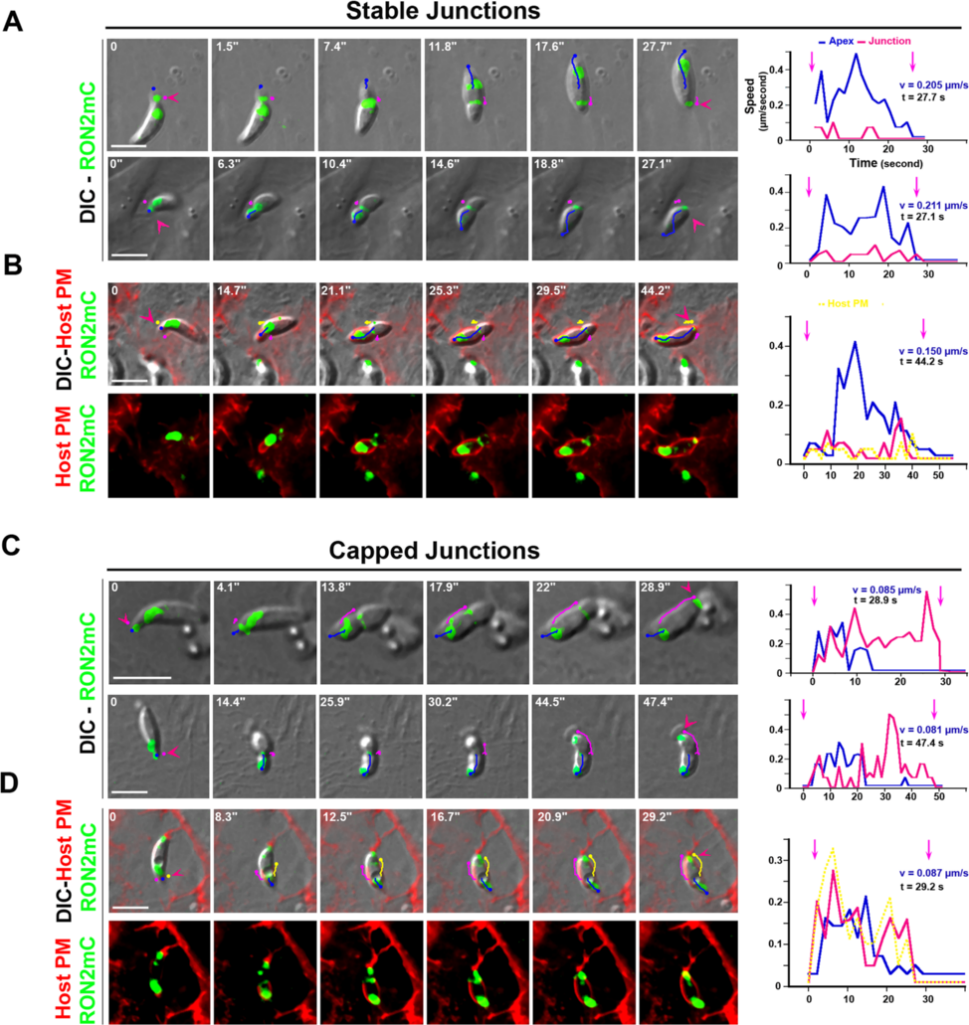
**RON2-expressing tachyzoites enter through a stable (A, B) or capped (C, D) junction.** DICmCherry (green) time lapses showing the penetration of tachyzoites into **(A)** HFF (top panel), HeLa (bottom panel), **(B)** Myr-Palm-GFP- HeLa cells (red), **(C)** Ptk-1 (top panel), HFF (bottom panel), **(D)** MyrPalm-GFP- HeLa (red); the blue and pink lines depict the trajectories of the parasite apex and the RON2- marked junction (green), respectively; pink arrowheads define the first and last signs of the junction; graphs on the right show the tachyzoite (blue) and the junction (pink) speeds over time; the PM movement during cell penetration is tracked with the yellow lines **(B, D)**; all scale bars: 5 μm. DIC, HeLa, human epithelial cervical cancer cells; HFF, human foreskin fibroblasts; Myr-PALM, myristoylated and palmitoylated; PM, plasma membrane; Ptk-1, rat kangaroo kidney epithelial cells; PV, parasitophorous vacuole; RON2, RhOptry Neck.

In order to analyze parasite motion after junction assembly, we tracked the spatial position (that is, xy coordinates) of both the parasite apex and the RON2mCherry-positive junction from the time of RON2 release into the host PM until the detection of a RON2 thick speck at the rear pole that typically precedes PV closure (Figure 3A,B, pink arrowheads). Analysis of the trajectories retrieved from the xy coordinates highlighted two situations: the first, and largely dominant one, when the tachyzoite continuously progressed into the forming PV to complete entry (Figure 3A, see the parasite apex trajectories in blue in time lapses, Additional file 1: Figure S1A), which was associated with a limited or no displacement of the RON2-labeled junction (that is, ‘stable or static junction’, see the junction trajectories in pink, Additional file 8: Movie 7). Graphs quantifying the parasite (blue line) and the junction (pink line) speeds for each time lapse attest the negligible speed of the junction during parasite penetration. As expected, when we precisely tracked the host PM movement at the entry site using host cells that express a fluorescent PM marker, we observed a full overlap with the RON2-labeled junction position over time (Figure 3B and the corresponding fluorescent panel, Additional file 6: Movie 5, the PM trajectory is depicted as a yellow line), thereby demonstrating that the latter was stably anchored in the PM and underlying complex. Aside from this scenario, we also observed that tachyzoite progression can be interrupted (Figure 3C,D, see parasite apex trajectories in blue) as assessed by the sharp drop in parasite speed (Figure 3C,D, see the corresponding graphs). Concomitantly, the junction coupled to the host PM was then capped backward (Figure 3C,D, see the junction trajectories in pink) with an increase in junction speed observed in the corresponding graphs. We have therefore referred to these events as ‘capped junction’ (see Additional file 9: Movie 8 and Additional file 10: Movie 9).

**Figure 4:**
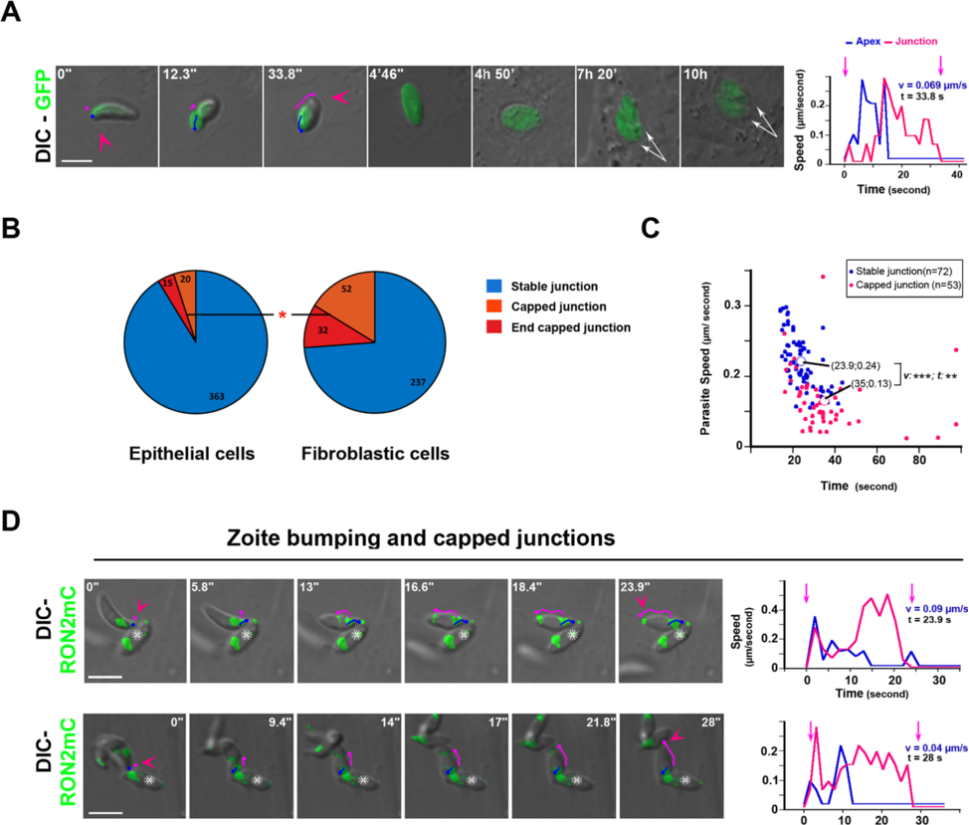
**Variation in the frequencies of stable versus capped junctions during cell entry by RON2mC-expressing tachyzoites. (A)** Time lapse of a GFP-tachyzoite using a capped junction to invade a NRK cell and further multiplying inside a competent PV, the parasite apex (blue line) and the junction (pink line) trajectories are shown, the pink arrowhead points to the junction site, white arrows mark the two daughter cells formed in the PV at about seven hours post-invasion; graph showing the tachyzoite (blue) and the junction (pink) speeds over time during the entry event with a capped junction. **(B)** Pie graphs showing the distribution of stable (blue), capped (orange) and end-capped (red) junctions in epithelial and fibroblastic cells. The absolute numbers are indicated; note the significantly higher frequency of all capping events in fibroblasts. **(C)** Scatter graph showing the average speed as a function of time for each stable (blue dots) or capped (pink dots) junction-associated event; note the highly significant differences in speed (v) and time (t) between the two types of junctions. **(D)** Time lapses showing tachyzoites that hit an already internalized parasite, which is marked with a white star during penetration into a HFF cell. The blue and pink lines reconstitute the trajectories of the parasite apex and the RON2-labeled junction (green) respectively; pink arrowheads define the first and last signs of the junction; graphs on the right show the tachyzoite (blue) and the junction (pink) speeds over time; note that the junction capping coincides with the time when parasites were forced to stop; all scale bars: 5 μm. HFF, human foreskin fibroblasts; NRK, normal rat kidney fibroblasts; PV, parasitophorous vacuole; RON2, RhOptry Neck.

Importantly, junction capping during entry led to parasite growth within the PV (Figure 4A). We next quantified in various cell types the amount of stable versus capped junctions and discriminated for the latter case, between early and late junction capping (referred to as ‘capped’ and ‘end capped’ junctions, respectively). Interestingly, we noticed a significant increase (*P* = 0.029) in the frequency of events with capped junctions when parasites invaded NRK and HFF fibroblasts as compared to HeLa and Ptk-1 epithelial cells (Figure 4B, the number of cases for each category is indicated) and the highest proportion was obtained with HFF cells. Consistently, kinetic analysis revealed that the average speed of the zoite during the internalization step was highly significantly lower for the capped junction than for the static junction, and the extent of reduction depended on the time at which the parasites were immobilized (Figure 3, Figure 4C (*P* <0.001)). Importantly, by carefully examining the kinematics of typical junction capping sequences, we validated the hypothesis that the average speed of zoite progression can be considered as ‘similar’ to the speed of junction retrograde movement (see Additional file 20: text information and corresponding graph), thereby arguing against a significant variation in the force produced during host cell entry in both situations. Collectively, these data strongly favor the idea that all parasites apply a mechanical force onto the junction regardless of the scenarios and suggest that the events of ‘junction capping’ correspond to situations of space constraints that hampered parasite forward progression and/or of weaker junction anchorage to the host cortex. In line with this scheme, the junction was immediately seen to translocate over the parasite body until the PM enclosed the zoite at the time the latter was forced to stop prematurely because of an obstacle such as a PV-containing parasite (indicated with a white star) (Figure 4D, see parasite and junction trajectories and corresponding graphs, Additional file 11: Movie 10).

### Kinematic modeling during cell penetration reveals typical changes in body curvature that are linked with two parasite rotations independently of junction scenario

**Figure 5:**
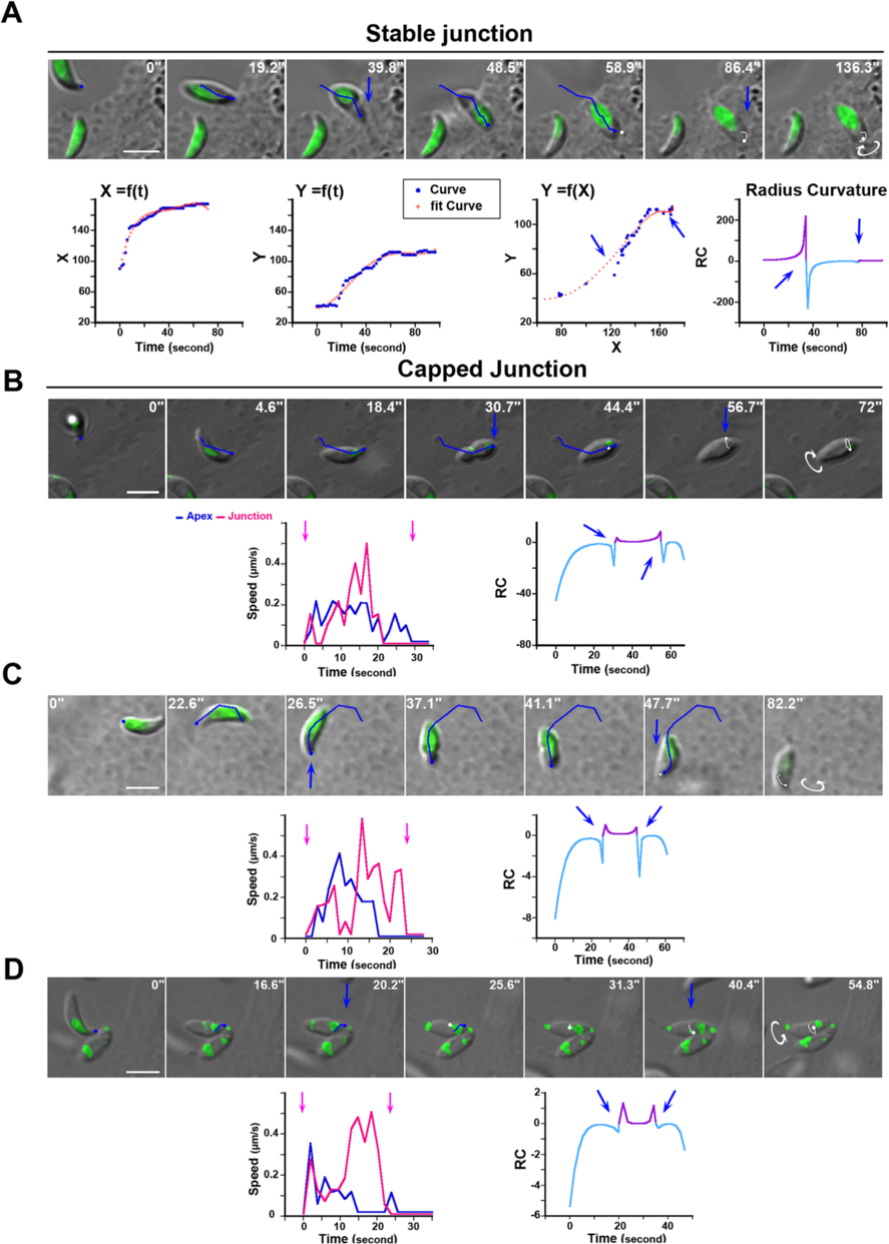
**Modeling of the tachyzoite trajectory during cell penetration highlights a change in body curvature and a final rotation independently of cell type and junction behavior.** Time lapses of: GFP tachyzoites entering in **(A)** a M2 cell with a stable junction, **(B)** RON2mC tachyzoite entering with a capped junction in a HFF cell, **(C)** a Ptk-1 cell with a capped junction, **(D)** Δ toxofilin RON2mC tachyzoite entering with a capped junction in a HFF cell. The lines define the parasite apex trajectory (blue line) and final rotation (white line); white curve arrows show the rotation direction while blue arrows indicate the change in body curvature, all scale bars: 5 μm. Below each time lapse is presented the corresponding graph: **(A)** of the x and y coordinates separately as a function of time, of the xy coordinates Y = f(X) that represents the tracked trajectory (blue), all with their respective best fitting polynomial curves (red crosses), and of the radius curvature (RC) as a function of time for which the change in line color matches with the sign of RC over time. The blue arrows mark the time of RC shift; **(B-D)** left graphs show the tachyzoite speed over time with parasite apex (blue lines) and junction (pink lines) trajectories, pink arrows indicate the starting and ending time points; right graphs display the RC as a function of time. HFF, human foreskin fibroblasts; Ptk-1, rat kangaroo kidney epithelial cell; RON2, RhOptry Neck.

Fluorescence emission in GFP-expressing tachyzoites appeared heterogeneous within the cell body revealing contrasted staining of sub-cellular vesicular like structures while fluorescent mCherry signals were restricted to rhoptries or pre-rhoptries. Working as reference points, these features provided a direct way to visualize the rotation of the parasite during entry that was initially described as a screw-like motion [3]. In order to study this motion, we manually tracked x and y Cartesian coordinates of the parasite apex during cell penetration (n = 174, 150 static and 24 capped junctions). Then we plotted each coordinate position (X = f(t) and Y = f(t)) and the xy trajectories (Y = f(X)) as functions of time and we searched for the best curve adjustment using the Matlab software (Figure 5A-D, see the corresponding graphs beneath the time lapse in panel A). For each invasion event, the curves typically showed two inflection points and were found best fitted by fourth degree polynomials (See Methods, Figure 5A and corresponding graphs on the bottom panel; the blue arrows mark the inflection points). Next, we computed the radius of curvature (RC) of the osculating circle to the xy trajectory at each time point because we observed that the zoite initiates entry with a circular movement and a given body curvature which changes while it rotates along its axis (Figure 5A, last graphs). A positive curvature described by an upwardly convex surface at the parasite specifies a given body curvature orientation whereas a negative one points to an upwardly concave surface that defines the other orientation, and the RC values describe the rotation by which the moving organism changes its direction. To better visualize the change in parasite curvature orientation, we assigned two different colors (that is, pale blue and purple) to the two opposite signs in the RC graphs and positioned the shift with blue arrows. While the trajectory analysis of events associated with a static junction revealed a shift in RC (blue arrow) that corresponds to the change of the curvature orientation accompanying the screw-like motion, another RC shift (blue arrow) was observed at the end of the trajectory and correlated with a second not yet identified rotation. This rotation exhibits different amplitudes depending on the event (white line and curved white arrows on the time lapses) and can be nicely observed with the change in plane of the rhoptry signal (Figure 5B, Additional file 12: Movie 11). This kinematic analysis of the tachyzoite motion clearly provides the description of the phases associated with entry until PV separation. Importantly, since the curvature shifts were detected not only when the parasite progresses through a static junction but also when it was stopped as a result of bumping into an obstacle or of facing denser cortex (Figure 5B-D, Additional file 13: Movie 12), we concluded that the same molecular mechanisms control the parasite contribution during entry with the static and capped scenarios.

### Deficient anchorage of the junction favors junction-PM capping but also leads to invasion failure, a situation exacerbated with parasites lacking the host cortical actin depolymerizer, toxofilin

**Figure 6:**
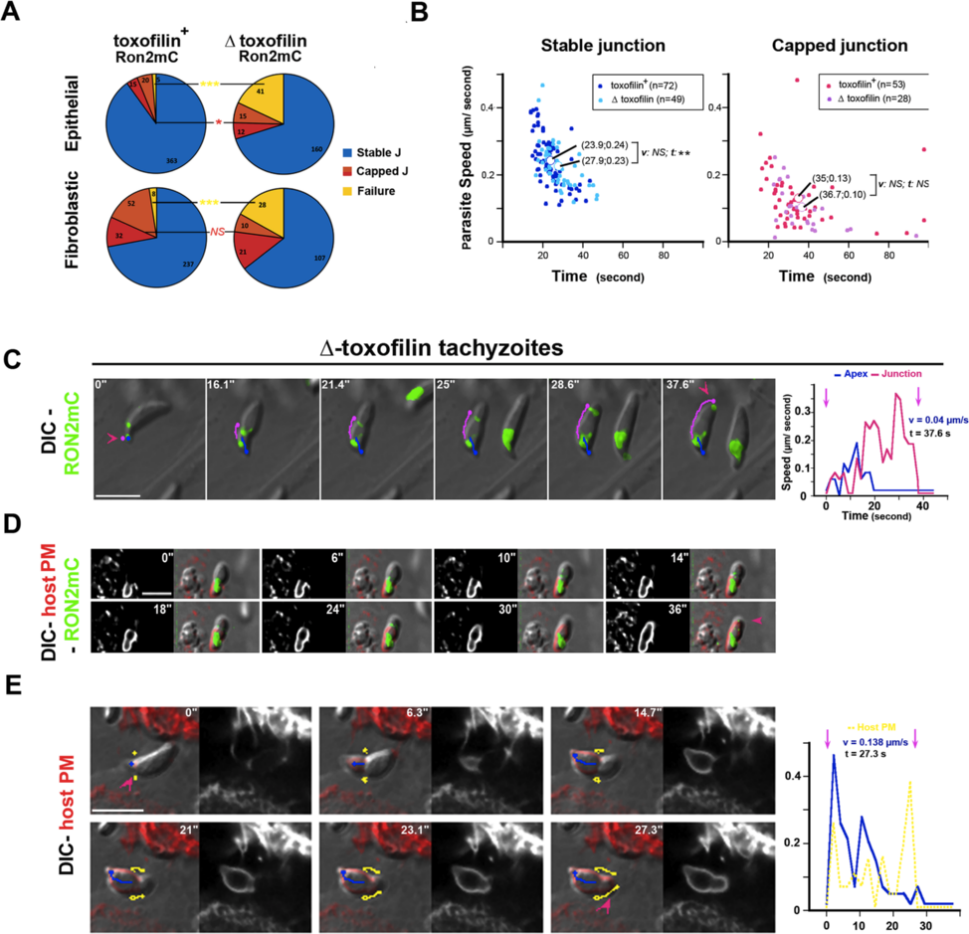
Δ **toxofilin parasites form more capped junctions than toxofilin** ^**+**^ **parasites. (A)** Pie graphs showing the distribution of stable (blue), capped (orange), end-capped junctions (red) and failed (yellow) events in epithelial and fibroblastic cells, the absolute numbers are indicated; note the increase in invasion failure when tachyzoites lack toxofilin and in capped junctions when they infect epithelial cells; **(B)** Scatter graphs showing the speed as a function of time for toxofilin^+^ RON2mC (dark dots) or Δ toxofilin RON2mC (pale dots) parasites and for stable (blue) or capped (pink) junctions; note the significantly faster process when the toxofilin^+^ tachyzoites enter with static junctions; **(C)** DIC-Cherry (green) merged time lapse showing the capping of the RON2-labeled junction during entry of a Δ toxofilin RON2mC parasite in a HFF cell; **(D)** DIC mC (green) merged time lapse showing a Δ toxofilin RON2mC parasite penetrating into a GFP-PH-PLCδ HeLa cell (red), the pink arrowhead shows the time when the parasite is enclosed in the host cell PM; **(E)** DIC-mCherry (green) merged time lapse showing Δ toxofilin RON2mC entering in a Myr-Palm-GFP expressing HeLa cell (red); pink arrowheads define the first and last signs of the junction; trajectories of: the parasite apex (blue line), the junction (pink line) and the PM (yellow line) are shown; graphs on the right show the tachyzoite (blue), the junction (pink) and the host cell PM (yellow) speeds over time; all scale bars: 5 μm; note that in **(E)** the tachyzoite creates a host cell PM evagination when moving forward and then translocates the host cell PM at the time it stops progressing, all scale bars: 5 μm. DIC, HeLa, human epithelial cervical cancer cells; HFF, human foreskin fibroblasts; Myr-Palm, myristoylated and palmitoylated; PM, plasma membrane; RON2, RhOptry Neck.

We have already shown that 1) the host cortical actin meshwork (CAM) is locally disassembled to create the appropriate space for tachyzoite progression and 2) *de novo* actin assembly is required for junction anchorage to the cortex [16]. We have proposed that the actin binding protein toxofilin [37], which is secreted at the site of entry and enhances actin turnover, promotes the rapid production of actin monomers that instantaneously fuel actin assembly that ensures junction anchorage [16]. Therefore, in the absence of toxofilin, we expected a weaker anchoring of the junction and, in turn, an increase in the frequency of penetration events associated with retrograde displacement of the junction. To test this hypothesis, we engineered parasites lacking the full coding sequence of toxofilin (Δ toxofilin) and carrying the RON2mCherry insertion at the endogenous locus (RHΔ*Ku80:*Δ*toxofilin:Ron2mCherry*), and we monitored the behavior of both the mutant and the parental toxofilin^+^ parasites. We found a significant increase in the number of junction capping events for the Δ toxofilin zoites when compared to the toxofilin^+^ only when entering in epithelial cells (n toxofilin^+^ = 35/398, n Δ toxofilin = 27/185, *P* = 0.026) (Figure 6A,C, Additional file 14: Movie 13). In the case of fibroblasts, these were already associated with a large proportion of capping events during entry of wild type parasites (Figure 4A). Movement of the host cell PM and junction capping were coupled as clearly seen in HeLa cells expressing a PH domain of *PLC*-δ fused to GFP that binds to PIP2 in the PM (Figure 6D). Moreover, analysis of kinetic parameters during penetration for each event showed that the duration (*P* = 0.66) and the zoite average speed (*P* = 0.13) did not significantly differ between wild type and mutant parasites for the capped junction scenario. Of note is the slightly longer process observed for the Δ toxofilin when compared to the toxofilin^+^ parasites (*P* = 0.046) in the case of the static junction scenario (Figure 6B). Crucially, junction and PM capping were also seen after the tachyzoite pushed the PM outward to create an evagination that further retracted in the cell cytoplasm as a *bona fide* PV (Figure 6E, Additional file 15: Movie 14). The corresponding graph shows the speed of the zoite (blue line) during forward progression and the increase in the host PM speed (yellow line) during junction capping.

**Figure 7:**
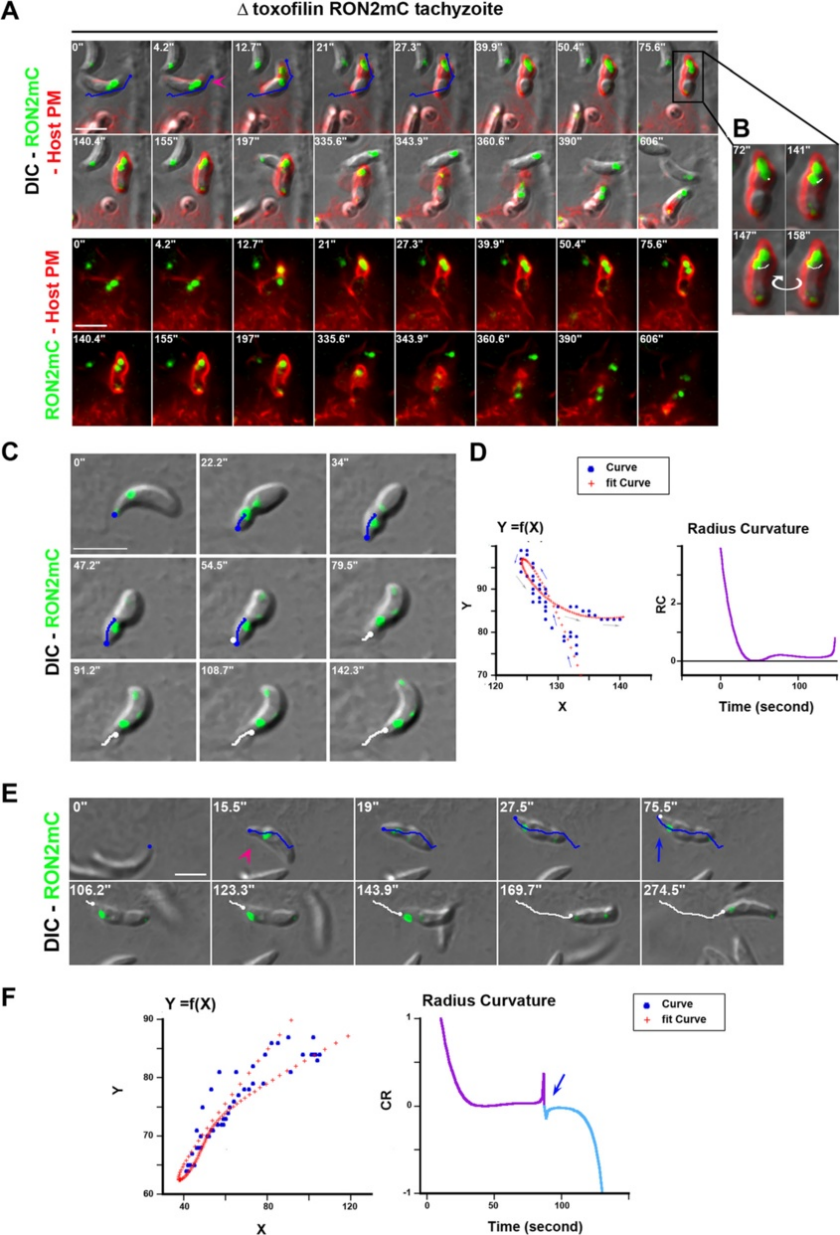
**Entry failure is drastically enhanced in Δ toxofilin parasites. (A)** Time lapses showing the initial engagement of the Δ toxofilin RON2mC parasite into the forming PV followed by their withdrawal. The two top panels show the DIC and fluorescent merged frames while the two bottom panels show the overlay between the parasite RON2mC (green) and HeLa cell Myr-Palm- GFP (red) signals; the pink arrowhead points to the start of entry and the blue line shows the initial forward trajectory of the tachyzoite. **(B)** Zoom from (A) on the time the tachyzoite rotates and starts to disengage, the white line follows the trajectory of the rhoptry-associated signal and the curved white arrow indicates the counter clockwise rotation. **(C)** Time lapse showing Δ toxofilin RON2mC parasite that releases RON2 into the PM and starts to penetrate (blue trajectory) but eventually withdraws (white trajectory) while RON2 moves backwards along the parasite surface. **(D)** Graphs corresponding to the time lapse (C) showing: the xy coordinates as a function of time (blue dots) and the fitting polynomial curve (red crosses), the path of the tachyzoite is labeled with arrows (blue = forward, grey = disengagement); and the radius curvature (RC) as a function of time. **(E)** Time lapse showing failure to complete entry while a faint signal of RON2mC is seen capped backwards at the posterior end of the tachyzoite, the pink arrowheads indicate the junction while parasite forward (blue) and backward (white) trajectories are drawn. **(F)** Graphs corresponding to the time lapse (E) showing: the xy coordinates as a function of time (blue dots) and the fitting polynomial curve (red crosses), and the radius curvature (RC) as a function of time; the RC shift is marked with a blue arrow.

**Figure 8:**
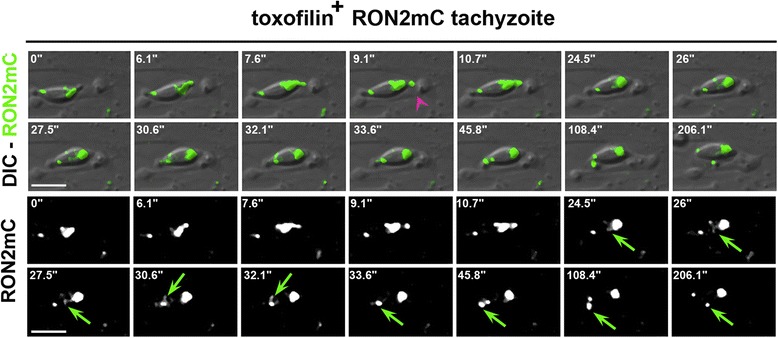
**Entry failure by a toxofilin** ^**+**^ **tachyzoite.** Time lapse of a toxofilin^+^ RON2mC parasite releasing RON2 to assemble a junction (pink arrowhead) into an HFF cell PM but then encountering a cell swelling area and failing to progress. A RON2 ring marked by a green arrow is concomitantly capped backwards, accumulates at the posterior and is subsequently released into the medium. The two top panels show the DIC and RON2mC (green) fluorescent merged frames while the two bottom panels show the parasite RON2mC signal; all scale bars: 5 μm. DIC, HFF, human foreskin fibroblast; PM, plasma membrane; RON2, RhOptry Neck.

In addition to the junction capping process that allows productive infection, in almost 20% of the events (n = 41/228 in epithelial cells and n = 28/167 in fibroblasts) we observed that the Δ toxofilin tachyzoites were unable to tract back the host cell PM once they were stopped in their progression. Instead, they disengaged within minutes with a counter clockwise rotation from the PM invagination that concomitantly resorbed leading to ‘invasion failure’ (Figure 7A, see zoom panel B, Additional file 16: Movie 15). Crucially, before parasites disengaged out of the PM inward fold, we observed rearward capping of the secreted RON2 material that accumulated as a posterior dot (Figures 7C and 8). Examination of the parasite trajectory during failure revealed a directional change of the parasite but no shift in body curvature until it got out (Figure 7D). However, few cases of a change in curvature before parasite withdrawal were observed and were always associated with a more advanced penetration process (Figure 7E). First, these findings strengthen the model of RON2 connected to the parasite motor and accordingly of a zoite force applied at the junction, thereby underpinning the latter as a traction site for force production. Secondly, they also strengthen the view that upon secretion into the host cell, toxofilin directs the fast coupling of actin disassembly at the CAM and of actin assembly at the junction. Finally, cases of invasion failures were rarely recorded with toxofilin^+^ parasites (n = 13/732, 1.8%) with a frequency significantly lower when compared to Δ toxofilin parasites (*P* = 0.016), and followed the same sequence as with the Δ toxofilin parasites (Figure 8). We showed here a tachyzoite with a piece of the residual body formed during intracellular replication [38] that remained attached at its posterior end which 1) displayed a typical helical pre-invasive gliding, 2) released RON2 molecules into the host cell PM and 3) attempted to penetrate into a region of the cell with strong swelling activity. While this tachyzoite was unable to overcome the local strong antagonistic pressure (that is, resistance) in the cell and, thus, to move forward, we could track the rearward capping of RON2 molecules and their trapping into the membranous piece of the residual body followed by the release of the whole material in the medium (Figure 8, Additional file 17: Movie 16). These data highlight the invasion failure as a consequence of insufficient anchorage of the RON complex to the host cell PM and the underlying cortex.

### Low cortex tension and high actin dynamics promote stable anchoring of the junction during entry by both toxofilin^+^ and toxofilin tachyzoites

**Figure 9:**
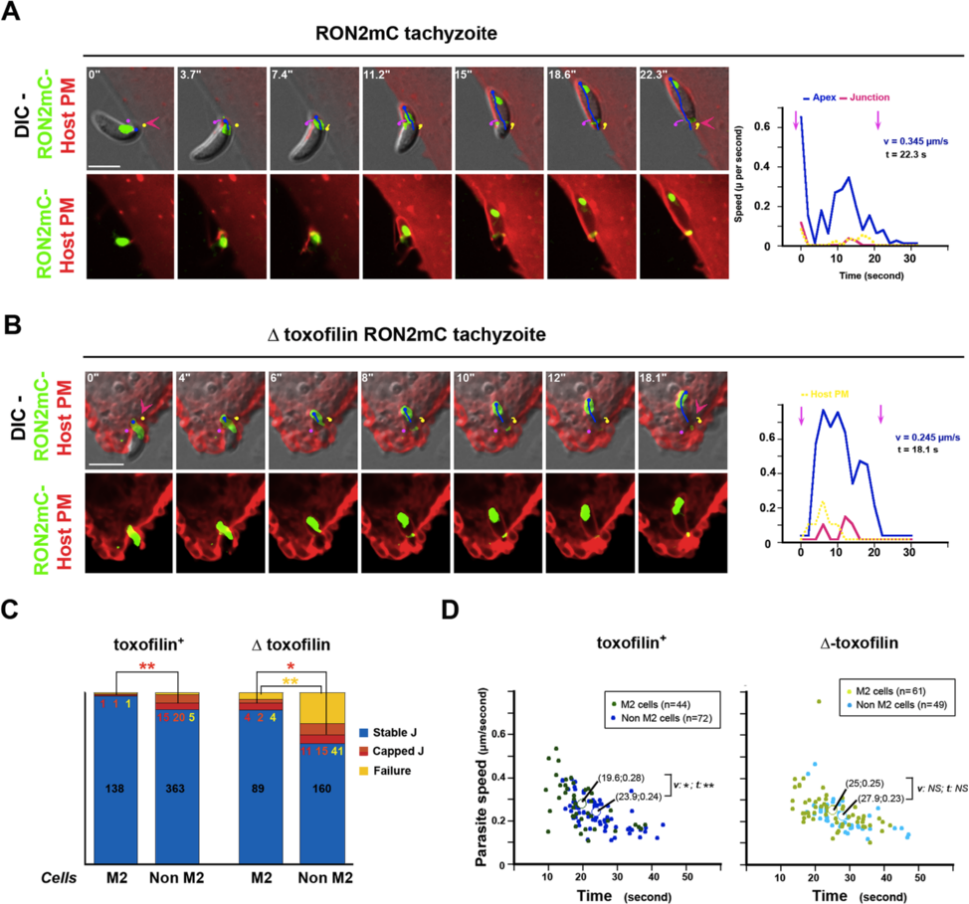
**Capped junctions and invasion failures are significantly reduced for both Toxofilin** ^**+**^ **and** Δ **toxofilin tachyzoites when entering in the** Δ **filamin A (M2) cells. (A, B)** Time lapses showing a toxofilin^+^ RON2mC **(A)** and a Δ toxofilin RON2mC **(B)** tachyzoite entering in M2 cells; the pink arrowheads define the first and last signs of the junction; DIC and mCherry (green) merged signals (top panels), RON2mC (green) and Myr-Palm-GFP (red) labeled host PM (bottom panels), the trajectories of the parasite apex (blue line), the junction (pink line) and the PM (yellow line) are shown; graphs on the right show the tachyzoite apex (blue), the junction (pink) and the host cell PM (yellow) speeds over time; all scale bars: 5 μm; **(C)** Histograms comparing the distribution of stable (blue), capped (orange), end-capped (red) junctions and failure (yellow) for toxofilin^+^ and Δ toxofilin tachyzoites when entering in M2 and non-M2 epithelial cells, the absolute numbers are indicated; note the prominence of stable junctions in all cases and the strong reduction in invasion failure, in particular for the mutant parasites; **(D)** Scatter graphs showing the average speed as a function of time for toxofilin^+^ RON2mC penetrating in M2 (green dots) and non-M2 (blue dots) cells and for Δ toxofilin RON2mC penetrating in M2 (pale green dots) and non-M2 (pale blue dots) cells; note the slightly significantly faster process when the toxofilin^+^ tachyzoites enter M2 cells. DIC, HFF, human foreskin fibroblast; Myr-Palm, myristoylated and palmitoylated; PM, plasma membrane; RON2, RhOptry Neck. (* p< 0.05, ** p< 0.01)

Since our data underline that junction capping and invasion failure were favored in cell regions of high cortex resistance and, in particular, when tachyzoites were genetically impaired in their ability to lower this resistance by disassembling actin filaments into monomers, we next hypothesized that in cells with low cortex tension and stiffness but with high amounts of cortical actin monomers, the parasites would have the greatest chances to smoothly move forward through an efficiently anchored junction into the forming PV. Additionally, if this proves correct, the lack of toxofilin should not translate in invasion failure. To test these assumptions, we performed a kinematic analysis of penetration events using M2 cells that do not express the cortex actin cross linker ABP-280 (that is, filamin A) and are consequently characterized by a low cortex tension and high cortical actin dynamics that translate into a high blebbing activity [39,40]. Remarkably, we found that after RON2 secretion and junction assembly into PM, the vast majoriy of toxofilin^+^ tachyzoites formed a junction that remained static (Figure 9A, Additional file 18: Movie 17) which correlated with a significant drop in the frequency of capping junctions when compared to non-M2 epithelial cells (Figure 9C, n M2 cells = 2/140 and n non-M2 cells = 35/398, *P* <0.01). Finally, this was also true in the case of Δ toxofilin parasites infecting M2 cells (n M2 cells = 6/95 and n non-M2 cells = 26 /186, *P* = 0.040) (Figure 9B,C, Additional file 19: Movie 18) but even more striking, was the significant reduction in the number of invasion failures (n M2 cells = 4/99 and n non-M2 cells = 41/227, *P* = 0.034) (Figure 7C). Of note, kinetic parameters showed a significantly shorter penetration process (*P* = 0.0017) with a slightly faster average speed (*P* = 0.04) when toxofilin^+^ tachyzoites infected M2 cells but this was not true for Δ toxofilin tachyzoites (Figure 9D). Collectively, these data reinforced the view that toxofilin acts by reducing cortex stiffness and consequently by promoting firm local anchoring of the junction to the host cell PM and cortex. Under this organization, the junction would be endowed with optimal mechanical properties to facilitate traction of the applied force.

## Discussion

While host-cell invasion is a vital process for most Apicomplexa parasites that has been explored for decades, the mechanistic model that prevailed all these years has recently shown its weaknesses and limitations. A primary issue concerns the relevance of the tight junction formed between the parasite and a permissive cell as a traction point for force transmission during cell invasion. We chose to re-examine this question using time-lapse imaging, tracking of the parasite apex and careful analysis of the kinematics of entry to quantitatively analyze invasion sequences under different parasite and host cell settings. First, we used *Toxoplasma* expressing a fluorescent version of RON2, the junction core component [12] reported as crucial for invasion [41,42]. RON2 inserts into the host cell PM and bridges the two cells during invasion: while it firmly binds to the zoite surface-exposed AMA1 protein, the role of this partnership has been controversial [23,42,43] and will only be resolved with a full understanding of the junction function(s). The parasites encoding *RON2mCherry* in replacement of *RON2* express a functional RON2 [22] and behave similarly as the parental strain, thereby enabling us to document dynamically that a burst of RON2 molecules traffics from the rhoptry to the tip of the extruded conoid, and subsequently inserts into the host cell plasma bilayer to initiate PV folding. Although RON2 trafficking was associated with parasite motion of a few μm/second, the RON2 insertion step into the host cell PM occurred when the parasite had almost stopped. Next, simultaneous tracking of the zoite apex and the RON2 secreted subset informed on the fate of the junction during the penetration phase. This kinematic analysis revealed that the vast majority of zoites typically progressed forward into the nascent PV by passing the whole body through the junction at an average speed of about 0.24 μm/second: in that situation, the junction’s spatial coordinates remained almost stationary, a feature consistent with a junction firmly anchored to the host cell-cortex and capable of sustaining a parasite motor propelling force. Moreover, analysis of the parasite trajectories using Maltlab software identified two changes in parasite body curvature in all penetration events, the second one ending the entry process. While the latter likely contributes to the fission process during PV separation, the first change in curvature corresponds to the screw-like behavior reported long ago [3], probably directed by the peculiar microtubule network organization of the zoite. These features again support an entry process driven by the parasite force(s).

An additional line of evidence for a propulsive force came from the visualization of junctions being capped towards the rear end of the tachyzoite in about 8% to 20% events. When we simultaneously used live-fluorescent labeling of the parasite and the host cell, we observed that the secreted RON2 molecular subset and the host cell PM rearward capping movement were coupled to eventually enclose the parasite in a growth permissive PV. In addition, kinematic analysis of the parasite motion showed that the changes in RC were also conserved in these events. These findings indicate that while the RON2 was properly organized within the lipid bilayer, the anchorage of the junction was weaker than for the static one and made the whole junction and cell PM more amenable to displacement while the zoite continued to pull. Indeed, the capping response precisely coincided with the arrest of the parasite on its way in, as exemplified by the tachyzoite hitting a PV-containing zoite. Finally, based on the duration of the penetration phase, and the fact that the sum of the speed of the parasite and of the capped junction was comparable during both scenarios, we propose that the zoite tracts onto the junction similarly in all events while premature immobilization allowed the visualization of the traction activity. Although it seems unlikely, the possibility theoretically remains that the capping of RON2 coupled to the cell PM would result from a still unknown host cell local response to the parasite-induced tension that would drag the junction and PM along the body to enclose the parasite without parasite motor requirement. However, this is even less likely if we consider the events where parasites progressed into the PM that did not invaginate but instead were pushed outwards (that is, by the parasite-exerting force), and thereby was not subjected to host cell viscosity/elasticity constraints. Crucially, such membrane evagination could be associated with junction and membrane capping, a situation only explained by the parasite pulling onto the membrane and a weaker anchorage of the junction (see Figure 6E and Additional file 15: Movie 14).

Local higher resistance beneath the PM commonly results from denser/thicker cytoskeleton lattices and primarily from the contractile cortical actin-myosin meshwork. Optimized Atomic Force Microscopy (AFM) has indeed revealed the spatial inhomogeneity of viscoelasticity over a cell [44]. These relate to regions of distinct membrane and cortical tensions providing specific nanomechanical properties, and defined by cortex thickness, actin dynamic, actin crosslinking and contractility [45]. Therefore, it is expected that, in the same cell, tachyzoites can penetrate by the classical forward progression and static junction or instead can trigger junction retrograde capping when they encounter a stiffer cortex area. In addition, we reported here that junction capping was more frequently observed in fibroblasts which display strong ventral and dorsal actin stress fibers that result in high membrane tension [46]. Of note, the highest rate of capped junction was repeatedly observed with the HFF cell line, which is the only growth limited cell line used throughout the study and, therefore, theoretically subject to aging. Aging is typically associated with an increase in cortex stiffening known to arise from an altered polymerization of the cortical actin cytoskeleton [47]. Conversely, in M2 cells that display a cortex of lower resistance since they lack the cortical actin cross-linker filamin, the entry with static junction was the rule including for toxofilin-deficient parasites (88.6%) that lack the ability to loosen a thick actin lattice [16]. Future studies on junction function will certainly gain from the increasing knowledge on membrane microdomains and nanoelastic properties at a single cell level. Of note, Coppens and Joiner elegantly proposed in the early 2000s that cholesterol in the host cell PM is necessary to trigger secretion of bulb rhoptry products that associate with cell entry [48]. The RONs discovery and their localization at the junction site [12,13] with the availability of the RON2mCherry strain now allow assessing in real time whether the host cell PM cholesterol is required for proper (1) secretion of the RON complex, (2) insertion of this complex into the host cell PM or (3) anchoring function of the junction.

Finally, key insights came from the observation of invasion failure, which while exceptional with wild type tachyzoites (less than 2%) is markedly increased in Δ toxofilin tachyzoites (up to 18%). While parasites were readily engaged in penetration, as judged by RON2 and PM folding, they failed to pull back the junction with the host cell PM after being stopped. Instead, they slowly disengaged from the cell and could twirl or pause again as free parasites. These new observations first contradict the current statement that RON2 assembly and the so-called junction structuring in the host cell PM commit parasite to invasion [42,49]. Secondly they clearly point to a role of toxofilin in providing the junction with the mechanical properties required for an efficient traction force. Indeed, the significant decrease in the amount of Δ toxofilin mutants that failed to invade M2 cells agrees well with the necessity of both cortical disassembly and free actin availability, provided through the toxofilin activity to complete invasion. Finally, when invasion aborted, a wave of RON2 molecules was capped towards the posterior end of the zoite where the material accumulated before being released in the medium. This is strongly suggestive of a motor engagement to the RON2-labeled junction on the parasite side. Whether a default in actin-mediated anchorage of the junction affects the stability of the RON2 ring in the cell bilayer inducing RON2 dislodgment upon force application awaits confirmation.

## Conclusions

This work provides a series of compelling evidence for the traction point property of the junction when the zoite applies a force, and is reminiscent of the focal adhesions acting as transmission sites for actomyosin-generated forces during cell motility onto a substrate. However, we are still missing the molecular identity of the functional junction to elucidate how force is exerted at this site. In this context, it will also be of future interest to dissect how the motor incompetent parasites can get access to the intracellular milieu including under constraints driven by three-dimensional tissue-like microenvironments, which strongly impact cell membrane and cortex mechanical properties. In addition, this work brings new tools to further investigate what is (are) the trigger(s) and the mechanisms responsible for RON2/RONs trafficking within the rhoptry neck and for their subsequent organization into the host cell lipid bilayer. The role of the conoid, a missing appendage in non-coccidian including *Plasmodium*, in the RONs organization during release and assembly should be examined in concert with the expected mechanical perturbations in the bilayer these should cause. Such changes in membrane and cortex tension might drive various local membrane responses at the micro-domain scale, some being, then, side effects of the entry process *per se*. Indeed, thin filopodia-like projections extending at the junction site along the tachyzoite were occasionally observed (see Additional file 18: Movie 17 and Figure 9A) although they differed from the membrane folds enwrapping the merozoite to provide force during red blood cell penetration [27]. It is noteworthy that *Plasmodium* zoites do not express toxofilin homologues but since they also face the cortical lattice of the host cell during penetration, they might use alternative strategies to overcome this constraint. Because the architecture of the red blood cell skeleton is quite different from those of nucleated cells, local targeting of a protein such as spectrin by the merozoite blood stage might be sufficient to trigger a cascade of events that leads to profound membrane and cortex rearrangements coincident with entry and underlying the characteristic echinocytosis process. Moreover, the slender hepatic stage of *Plasmodium* (that is, the sporozoite) might not need to loosen the cell cortex to the extent that the *Toxoplasma* bulky tachyzoite does, or/and might use different pathways to this end, such as proteolysis of key actin-membrane adaptors. Therefore, future comparative studies on the mechanisms by which *Plasmodium sp.* and *Toxoplasma* zoites infect host cells will undoubtedly highlight similarities and singularities in the fine tuning of the invasion strategies selected by the two parasites.

## Methods

### Cells and parasites

All media and products used for cell culture were from Gibco-Life technologies (St Aubin, France). HFF, NRK and HeLa were grown in (Dulbecco’s) modified Eagle’s medium ((D)MEM) supplemented with glutamax (Gibco), heat-inactivated FCS (10%), penicillin (100 U/ml), streptomycin (100 mg/ml) and 10 mM HEPES. PtK1 were cultured in Ham’s F12-medium (Sigma-Aldrich, L'isle d'Abeau Chesnes, St Quentin Fallavier, France) containing 25 mM HEPES, 10% FBS and antibiotics. All cultures were maintained at 37°C and 5% CO^2^ atmosphere. *T. gondii* strains (RH-GFP, RH*-*Δ*Ku80-RON2mCherry*, RH*-*Δ*Ku80-*Δ*toxofilin-RON2mCherry*) were propagated on HFF cells as described [50]. To characterize the *RH-*Δ*Ku80-RON2mCherry* strain, we compared (1) the pre-invasive trajectories and (2) the duration of the invasion event of RON2-tachyzoites and RON2mCherry using the techniques described in the section below referring to image analysis. In addition, using laser scanning confocal microscopy, we quantified the progeny per vacuole (n = 500 vacuoles in triplicates per assay, two independent growth assays) of the two strains developing on a HFF monolayer after 30 minutes of contact followed by extensive washing of the culture to remove extracellular parasites and by 28 hours of culturing.

### Molecular cloning, transfection and cell line selection

#### T. gondii

The RHΔ*Ku80:*Δ*toxofilin:Ron2mCherry* strain was generated by first replacing the endogenous *toxofilin* locus with the *hxgprt* gene by homologous recombination. The Multisite Gateway Pro 3-fragment Recombination system was used to clone the HXGPRT cassette (amplified with primers HX-B4r:

- GGGGACAACTTTTCTATACAAAGTTGCAGCACGA
- AACCTTGCATTCAAACCCG and HX-B3r: GGGGACAACTTTATTATACAAAGTTGT
- GATCCCCCTCCACCGCGGTGTCACTG) flanked by the 1 kb 5′(attB1toxo: GGGGACAA
- GTTTGTACAAAAAAGCAGGCTGGTACCACGAGCACAGCCGACTGGCAC/attB4toxo:
- GGGG AC AAC TTTGTATAGAAAAGTTGGGTGGTTCGACGCGTCGACGCCT) and the 1 kb 3′ (attB3toxo: GGGGACAACTTTGTATAATAAAGTTGCGAATCTGTTTGGGAT
- GGCTTTGAC/ attB2toxo: GGGGACCACTTTGTACAAGAAAGCTGGGTATGTAGGGT

TCCACTGTCCTGCGG) up and downstream the *toxofilin* coding sequence. The target sequence was amplified (Phusion High Fidelity DNA polymerase, NEB, Ipswich, MA, USA) and 10^7^ parasites were electroporated with 25 μg of the PCR product. Recombinant parasites were selected with 25 μg/ml mycophenolic acid and 50 μg/ml xanthine and cloned by limiting dilution. Then, the *ron2* gene was fused to the *mcherry* coding sequence as described in [22] except that the 1.1 kb fragment corresponding to the 3’end of the *RON2* gene was cloned into the mcherry-LIC-DHFTRS vector. Clones were selected with 500 ng/ml pyrimethamine and cloned by limiting dilution.

#### Mammalian cells

Generation of CAAX-mCherry retroviruses is described in [51]. Generation of PHPLCΔ-GFP retroviruses is described in [52]. To generate cells stably expressing a GFP tagged membrane marker, we excised MyrPalm from a plasmid acquired from MyrPalm-CFP (plasmid 14867, [53]) and inserted it into pRetroQAcGFPC1. Retroviruses were then generated by transfecting the plasmids into 293-GPG cells for packaging (a kind gift from Prof. D. Ory, Washington University) [54]. For generation of stable cell lines, retroviral supernatants were used to infect wild type HeLa cells or M2 cells. The cells were selected in the presence of 500 ng/ml puromycin or 1 μg/ml G418 for two weeks.

### Videomicroscopy and image acquisition

Parasites were collected within a few hours following spontaneous egress from the HFF monolayers and washed in HBSS supplemented with 1% FCS. Time-lapse video microscopy was conducted in chamlide chambers (LCI Corp., Seoul, Korea) installed on an Eclipse Ti inverted confocal microscope (Nikon France Instruments, Champigny sur Marne, France) with a temperature and CO_2_-controlled stage and chamber (LCI Corp., Seoul, Korea), equipped with a coolsnap HQ2 camera (Photometrics, Roper Scientific, Lisses, France) and a CSU X1 spinning disk (Yokogawa, Roper Scientific, Lisses, France). The microscope was piloted using Metamorph software (Universal Imaging Corporation, Roper Scientific, Lisses, France) and images were acquired with settings including 1 frame/second for 20 minutes, with one to two laser wavelengths depending on the experiment.

### Image analysis

Images stacks for every event of interest (pre-invasive motion, cell penetration, entry failure, and so on) were prepared and annotated with time, scale and arrows with Metamorph software from the raw image data file. Next ImageJ software (Rasband, W.S., ImageJ, US National Institutes of Health, Bethesda, MD, USA [55], 1997–2014). and the ‘manual tracking’ plugin were used to simultaneously track the spatial positions of the parasite’s apex, the RON2 fluorescently labeled junction and the fluorescently labeled host cell PM at the junction over time. The xy Cartesian coordinates allowed reconstituting trajectories of interest. Saving these data as Excel files and integrating the time interval between consecutive frames provided both the instantaneous and mean speeds of the parasite of interest. Retrieved data of the instantaneous velocities and the frames they referred to were next imported to KaleidaGraph Software (Synergy Software, Reading, PA, USA) to plot the parasite’s speed as a function of time.

### Kinematic analysis of the parasite trajectory during cell penetration

In order to describe the kinematic motion of the tachyzoite in mathematical terms, the Cartesian coordinates system was chosen for being the most adapted to data fitting. The different x and y coordinates which were manually tracked with ImageJ were plotted as a function of time before being fitted by fourth degree polynomials. Fourth degree polynomials were chosen over third degree polynomials for their convenience in systematically fitting the trajectory curves with two inflection points, a feature that appeared characteristic of tachyzoite invasive motions. Homemade Matlab routines were used to derivate these polynomials twice to obtain x’(t), y’(t) and x”(t), y”(t) thereby allowing to assess the RC of the osculating circle along the curve defining the trajectory at each time point. The RC at a given point of this curve corresponds to the radius of a circle that is the most tangent (the osculating circle, see the diagram below with the change in the RC values) and mathematically best fits the curve at that point. It is defined as the osculating circle also called the curvature center of the curve. RC values were obtained by the formula:

$$ RC(t)=\frac{{\left(x\hbox{'}{(t)}^2+y\hbox{'}{(t)}^2\right)}^{3/2}}{y\hbox{'}\hbox{'}(t)x\hbox{'}(t)-x\hbox{'}\hbox{'}(t)y\hbox{'}(t)} $$

Among the 150 cases showing a static junction, 24 and 27 were recorded on HFF and NRK fibroblasts, respectively, and 11, 36 and 52 on HeLa, M2 and Ptk-1 epithelial cells, respectively. With regard to the capped junction events, three and four were obtained on NRK and HFF fibroblasts, respectively, while two, four and eleven were observed on M2, HeLa and Ptk-1 epithelial cells, respectively.

### Immunofluorescence labeling of invading tachyzoites

To catch parasites in the process of penetrating into HeLa cells, we performed invasion assays as previously described [9]. After paraformaldehyde fixation (2% in PBS, 30 minutes), free aldehydes were quenched in NH4Cl (50 mM, 10 minutes), and cells were incubated in blocking buffer (2% BSA in PBS, 30 minutes), then with anti-P30 antibodies (20 minutes, 23°C) (Novocastra, Nanterre, France) followed by Alexa Fluor*®* 660 conjugated secondary antibodies (30 minutes, 23°C) (Molecular Probes, Life Technologies, St Aubin, France). Samples were next permeabilized with 0.5% Triton-X 100 (five minutes, 23°C), exposed again to the blocking buffer and incubated with Alexa Fluor*®* 488 conjugated phalloidin to stain F-actin (45 minutes, 23°C). In other assays, cell permeabilization was performed after fixation and affinity-purified anti-RON4 antibodies were used before Alexa Fluor*®* 488 conjugated secondary antibodies. Cells were mounted in Mowiol® 4–88 (Sigma Aldrich, St Louis, MO, USA) and analyzed by confocal microscopy using the Eclipe Ti inverted microscope.

## Additional files

Additional file 1: Figure S1. RON2-expressing tachyzoites enter through a stable (A) or capped (B) junction. Additional file 2:
**Movie 1 Impulse motion of a GFP-tachyzoite on top of Ptk-1 cells that precedes entry.** The blue line defines the trajectory and the pink arrowhead marks the constriction that is expected to represent the junction. Additional file 3:
**Movie 2 Circular gliding of a GFP-tachyzoite on top of NRK cells that precedes entry.** The blue line defines the trajectory and the pink arrowhead marks the constriction that is expected to represent the junction. Additional file 4:
**Movie 3 Helical gliding of a GFP-tachyzoite on top of Ptk-1 cells that precedes entry.** The blue line defines the trajectory and the pink arrowhead marks the constriction that is expected to represent the junction. Additional file 5:
**Movie 4 Twirling motion of a GFP-tachyzoite on top of M2 cells that precedes entry.** The blue line defines the trajectory and the pink arrowhead marks the constriction that is expected to represent the junction. Additional file 6:
**Movie 5 A RON2mC-tachyzoite 1) secretes a burst of Ron2mC molecules that assemble as junction into the MyrPalm-GFP positive PM of a HeLa cell and 2) penetrate in to a MyrPalm-GFP positive PM with a static junction.** The pink arrowhead marks the RON2-labeled junction. Additional file 7:
**Movie 6 Two RON2mC-tachyzoites that display a helical gliding on top of HFF cells; one (1) secretes RON2mC and form the junction before penetration into the cell while the other (2) does not spit RON2mC and pauses as extracellular.** The pink arrowhead marks the RON2-labeled junction. Additional file 8:
**Movie 7 A Ron2mC-tachyzoite penetrates with a static junction into a HFF cell.** The pink arrowhead marks the RON2-labeled junction. Additional file 9:
**Movie 8 A RON2mC-tachyzoite penetrates with a capped junction into a Ptk-1 cell.** The pink arrowhead marks the RON2-labeled junction. Additional file 10:
**Movie 9 A RON2mC-tachyzoite penetrates with a capped junction into a HeLa cell expressing MyrPalm-GFP at the plasma membrane.** The pink arrowhead marks the RON2-labeled junction. Additional file 11:
**Movie 10 A RON2mC-tachyzoite hits a vacuole-containing parasite (indicated with a white star) in the course of entry into a HFF cell, which causes its junction to be capped at the posterior end.** The pink arrowhead marks the RON2-labeled junction. Additional file 12:
**Movie 11 GFP-tachyzoite penetrates with a stable junction into a M2 cell.**
Additional file 13:
**Movie 12 A RON2mC-tachyzoite penetrates with a end-capped junction and a final rotation into a HFF cell.**
Additional file 14:
**Movie 13 A toxofilin RON2mC-tachyzoite penetrates with a capped junction into a HFF cell.** The pink arrowhead marks the RON2-labeled junction. Additional file 15:
**Movie 14 A toxofilin RON2mC-tachyzoite moves forward into a HeLa cell expressing MyrPalm-GFP at the host cell PM and creates an evagination, then capped the junction and the PM to complete invasion.** The pink arrowhead marks the RON2-labeled junction. Additional file 16:
**Movie 15 A toxofilin RON2mC-tachyzoite failed to enter into a HeLa cell expressing MyrPalm-GFP at host cell PM.** The pink arrowhead transiently marks the sign of an early junction. Note the rotation visualized by the rhoptries displacement prior to disengagement out of the cell PM invagination. Additional file 17:
**Movie 16 A toxofilin** ^**+**^ **and RON2mC-tachyzoite fails to enter into a HFF cell and capped RON2 molecules backwards which are subsequently released into the medium.** The pink arrowhead transiently marks the sign of an early junction. Additional file 18:
**Movie 17 A toxofilin and RON2mC-tachyzoite enters with a stable junction into a M2 cell.** The pink arrowhead marks the RON2-labeled junction. Additional file 19:
**Movie 18 A toxofilin and RON2mC- tachyzoite enters with a stable junction into M2 cell expressing MyrPalm-GFP at the plasma membrane.** The pink arrowhead marks the RON2-labeled junction. Additional file 20:
**Text Tachyzoite speed during forward progression and junction speed during capping.**
