# Supplementary figures and images for: The toxoplasma-host cell junction is anchored to the cell cortex to sustain parasite invasive force
Source: BMC Biol. 2014 Dec 31;12:773. doi: 10.1186/s12915-014-0108-y (PMC4316648; doi:10.1186/s12915-014-0108-y)

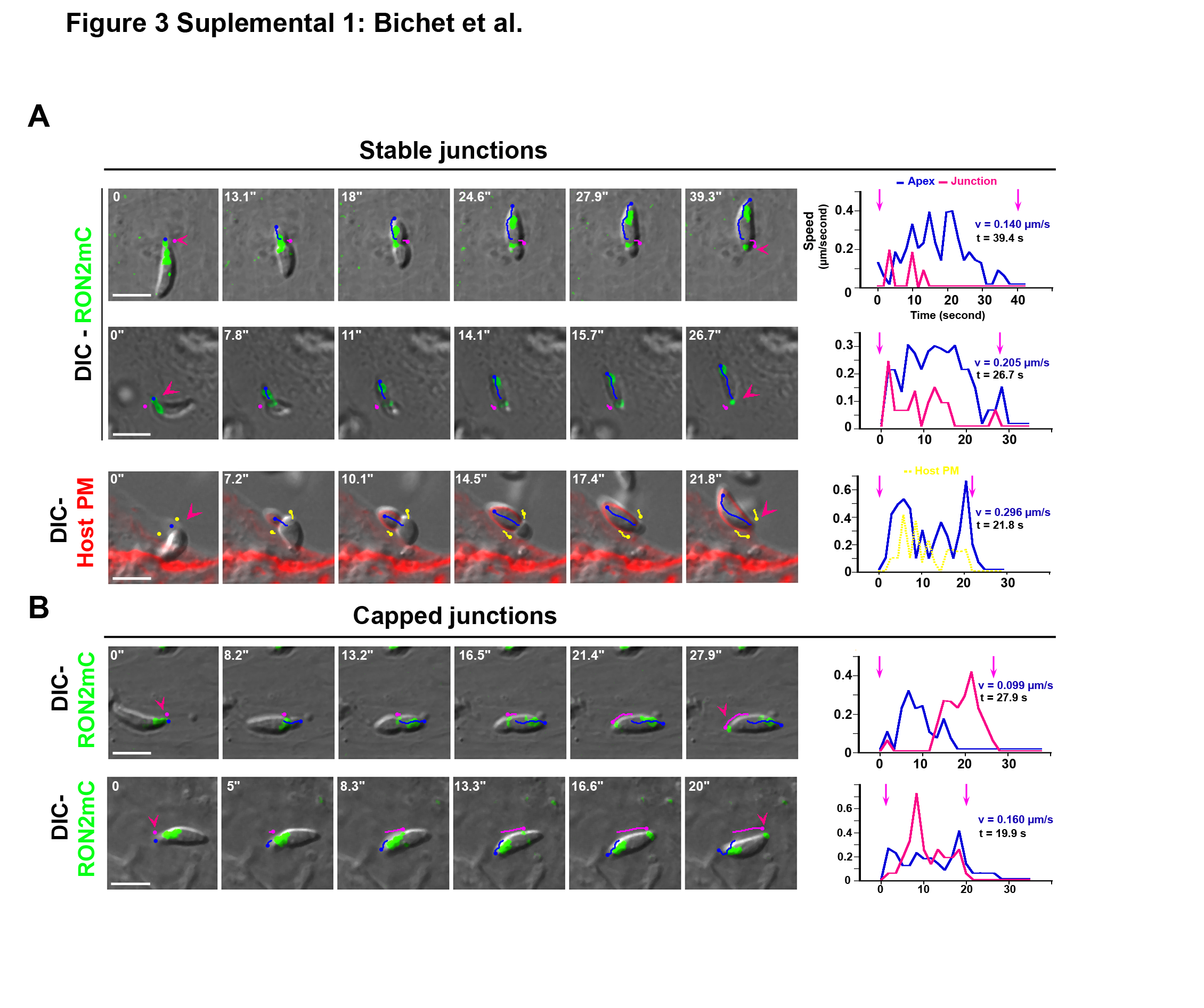

Supplement: Additional file 1: Figure S1. — RON2-expressing tachyzoites enter through a stable (A) or capped (B) junction. [file 12915_2014_108_MOESM1_ESM.tiff]
