# Supplementary material for: The toxoplasma-host cell junction is anchored to the cell cortex to sustain parasite invasive force
Source: BMC Biol. 2014 Dec 31;12:773. doi: 10.1186/s12915-014-0108-y (PMC4316648; doi:10.1186/s12915-014-0108-y)

# Additional file Text

**Tachyzoite speed during forward progression and junction speed during capping**

Even though the speed and the duration of the penetration process vary in every event, a successful invasion implies a distance to be covered, either by the parasite (i.e. stable junction) or the junction (i.e. capped junction). In the latter situation, when the capping started about midway (see the graph below as an example), two successive parts can basically be distinguished during the penetration event. In the first, the parasite apex moves into the host cell, as the junction remains static while in the second, the parasite apex is stopped and the junction starts moving backwards, achieving the internalization process. We hypothesized that the intensity of the force applied by the parasite or the junction remains about constant throughout the process. To test this hypothesis, we integrated the instantaneous speed (response of the junction and the parasite when in motion) on a two time phases, therefore the figures obtained were homogeneous to a distance. We found that, when the two phases are clearly separated, the portions of the total time spent to move are nearly similar to the portion of the total distance covered (for the parasite as for the junction), which validates the initial hypothesis and agrees with a conserved force process all along the event


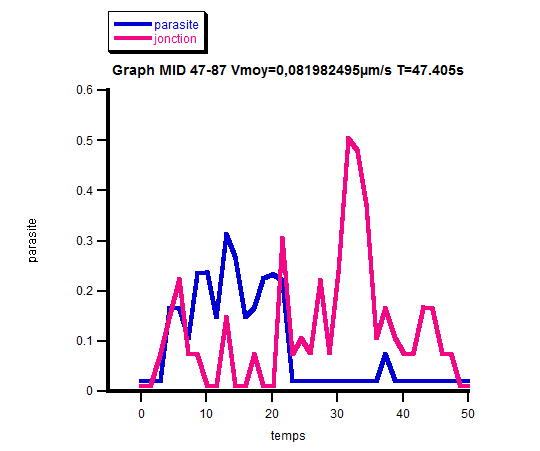





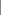

Supplement: Additional file 20: — Text Tachyzoite speed during forward progression and junction speed during capping. [file 12915_2014_108_MOESM20_ESM.docx]
